# Optimal timing of interictal FDG‐PET for epilepsy surgery: A systematic review on time since last seizure

**DOI:** 10.1002/epi4.12617

**Published:** 2022-06-20

**Authors:** Nienke N. de Laat, Nelleke Tolboom, Frans S. S. Leijten

**Affiliations:** ^1^ Utrecht University Utrecht The Netherlands; ^2^ Department of Radiology and Nuclear Medicine University Medical Center Utrecht Utrecht The Netherlands; ^3^ Department of Neurology and Neurosurgery University Medical Center Utrecht Utrecht The Netherlands

**Keywords:** F‐fluorodeoxyglucose (18F‐FDG), glucose metabolism, positron emission tomography (PET), postictal, temporal lobe epilepsy (TLE)

## Abstract

Interictal 18F‐Fluorodeoxyglucose positron emission tomography (FDG‐PET) is used in the workup for epilepsy surgery when MRI and EEG video monitoring are not conclusive. Timing of FDG‐PET is crucial to avoid the metabolically dynamic (post)ictal state that complicates interpretation, but the exact time window is unclear. We performed a systematic review to provide an evidence‐based recommendation for the minimal time interval between last seizure and FDG‐PET acquisition. We searched PubMed and Embase for articles on the effect of time since last seizure on FDG‐PET outcome. Quality assessment was conducted with the Critical Appraisal Skills Programme Cohort Study Checklist. We identified five studies. Three studies were classified as of low to moderate quality, mainly due to undocumented data or insufficient statistical measurements. Two high‐quality studies included only adults with Temporal Lobe Epilepsy (TLE). The metabolic interictal phase is 24 or 48 hours after the last seizure, depending on seizure type. The recommendation is based on the best available evidence from two small study populations for TLE. If clinically possible, interictal FDG‐PET in adults should be performed at least 24 hours after focal aware seizures and 48 hours after focal impaired awareness and focal to bilateral tonic–clonic seizures.

## 1 INTRODUCTION

In focal intractable epilepsy, resective surgery can be considered. In most presurgical evaluation protocols, the epileptic focus is localized with magnetic resonance imaging (MRI), routine electroencephalogram (EEG), and EEG video monitoring (EVM). When these tests are inconclusive, an interictal 18F‐Fluorodeoxyglucose positron emission tomography (FDG‐PET) is recommended.^1^, ^2^, ^3^ Timing is crucial to avoid the metabolically dynamical postictal state that complicates interpretation. The epileptic focus is hypometabolic in the interictal phase and may be hypermetabolic in the ictal and postictal phase.^4^, ^5^, ^6^, ^7^

The start of the postictal state and especially the postictal–interictal transition is clinically challenging.^8^ The postictal state has recently been defined as “a temporary brain condition following seizures lasting minutes to days”.^9^ During and after the ictal phase, FDG‐PET may show the focus to be hypermetabolic.^5^, ^10^, ^11^ This may lead to false lateralization if FDG‐PET was supposed to be interictal and hypometabolism relative to the contralateral side is the basis of interpretation. When ictal FDG‐PET was intentionally studied,^12^ complex dynamic patterns were observed, probably because FDG uptake and FDG‐PET acquisition exceed the average time of a seizure. This is further complicated by ictal movements that increase uptake in motor areas. In the postictal phase, the pattern depends on the timing of injection after seizure.^13^

**FIGURE 1:**
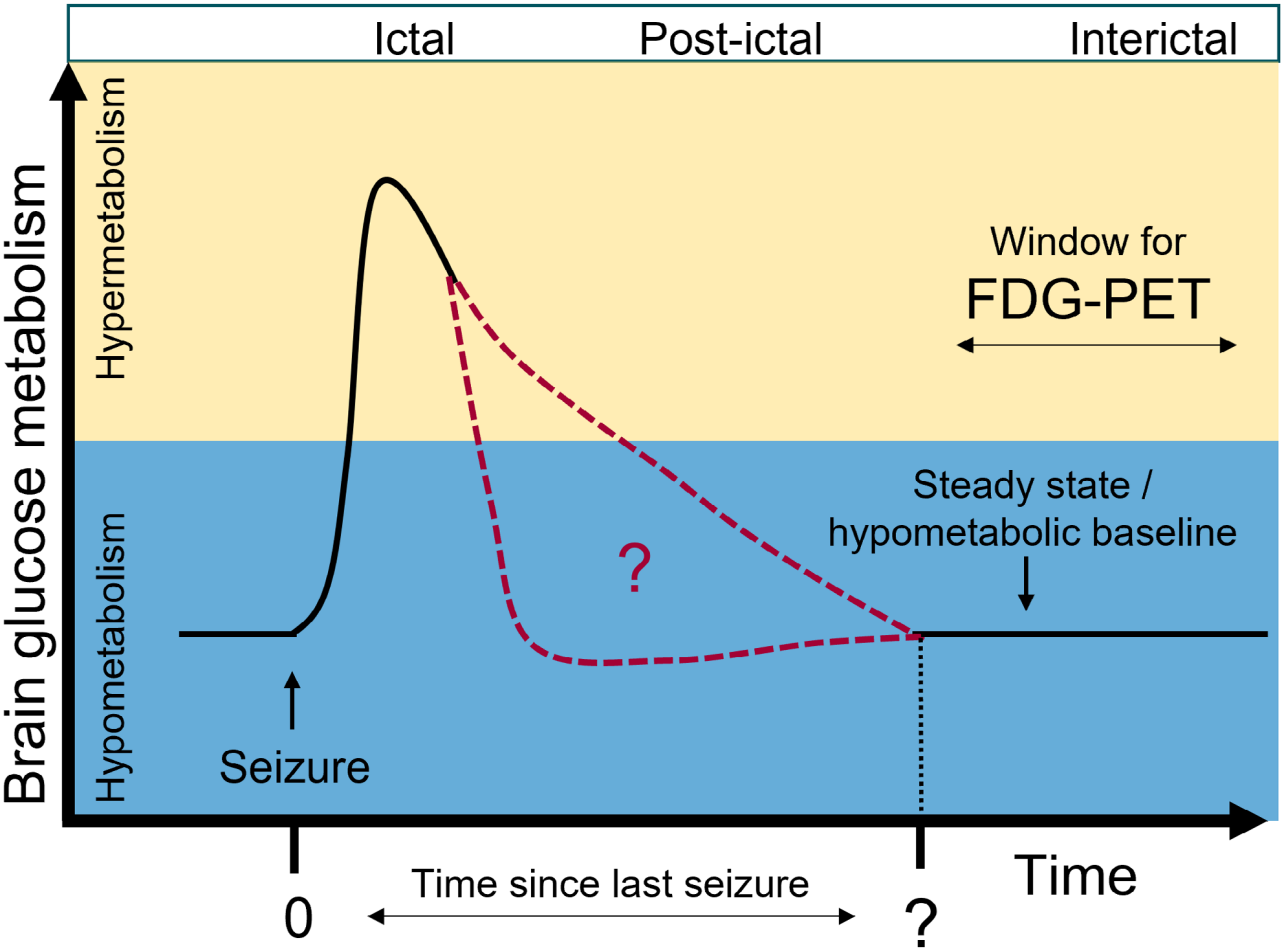
Graphic conceptualization of dynamic brain glucose metabolism following an epileptic seizure (time = 0) in epileptogenic foci. The interictal hypometabolic (blue area) baseline is disturbed by a seizure resulting in temporary hypermetabolism (yellow area). The progression of the brain glucose metabolism is unknown (red dotted line) as is the time since last seizure to the hypometabolic steady state. Abbreviation: FDG‐PET, fluoro‐2‐deoxyglucose positron emission tomography

At which point the postictal phase becomes metabolically interictal is unknown^14^; see Figure 1. This image illustrates “time since last seizure” which refers to the time interval between the last seizure and FDG‐PET imaging. Only in the interictal phase, that is, in the normalized steady state between epileptic seizures, FDG‐PET reliably shows focal hypometabolism in epileptic foci,^4^ explained by a variety of mechanisms including reduction in synaptic density and neuronal loss.

It is therefore clinically important to define the hypometabolically stable interictal state. The aim of this systematic review is to evaluate the evidence and provide a recommendation for timing of FDG‐PET.

## 2 METHOD

This systematic review is reported following the Preferred Reporting Items for Systemic Reviews and Meta‐Analyses (PRISMA) guidelines. The study protocol was registered with the Prospero international prospective register of systematic reviews (Prospero ID: CRD42021227699). The strategy of this review was to (1) identify all relevant research, (2) asses the quality of the literature, (3) systematically synthesize the relevant findings, (4) critically appraisal the included studies, and (5) formulate a recommendation.

### 2.1 Search strategy

The following Mesh Terms were used in PubMed: epilepsy, seizures, fluorodeoxyglucose F18, and brain/metabolism. No filter was applied, the search was not constrained on publication year. Used Emtree terms in Embase were as follows: epilepsy, seizure, Fluorodeoxyglucose F18, and brain metabolism. For all terms, different spelling and synonyms were used. In Embase, the “sources filter” was applied to only include Embase results and exclude Medline because they overlap with the results in PubMed. The detailed search strategy is found in Appendix S1.

### 2.2 Eligibility criteria

Online databases PubMed and Embase were searched on 5th of May, 2022. We included the studies of epilepsy patients which reported time since last seizure, and imaging with 18F FDG‐PET. Conference abstracts and studies without novel data collection and were excluded. We did not limit eligibility by publication year or study design.

### 2.3 Quality assessment

We assessed all papers for quality using the Critical Appraisal Skills Programme (CASP) Cohort Study Checklist.^15^ All studies included were observational cohort studies.^16^ Their quality is presented in percentages.

### 2.4 Data extraction and analysis

The following study characteristics were extracted: first author, year of publication, study design, number of inclusions, patient demographics, seizure type, time points, and FDG‐PET analysis. The data were analyzed descriptively. Data collection focused only on time since last seizure. Seizure type is reported according to the new classification by the International League Against Epilepsy (ILAE). Studies scoring higher than 60% on quality were used for the recommendation.

## 3 RESULTS

### 3.1 Study selection

The search strings provided 83 and 70 results in PubMed and Embase, respectively (for flow diagram, see Figure S2). Search results were merged using reference management software Rayyan. Animal and pharmacological studies were excluded on title alone; from other articles full abstract were read. There were multiple articles about interictal FDG‐PET, but only 11 articles included time since last seizure. Five articles met inclusion criteria and were included in this systematic review.

### 3.2 Data synthesis

The aim, statistical method, results, conclusions, and CAPS score of these five studies^14^, ^17^, ^18^, ^19^, ^20^ are summarized in Table 1. The study data are shown in Table S3. Publication year ranged from 1994 to 2013. Most studies were retrospective^17^, ^18^, ^19^, ^20^ except one.^14^ Two studies included children.^18^, ^19^ Three of the studies included adult patients with temporal lobe epilepsy (TLE).^14^, ^17^, ^20^ All studies had different measurements for time since last seizure. The outcome, determined by analysis of FDG‐PET images, was defined differently in each study. This is explained in Appendix S4.

**TABLE 1:** Data collection of included studies. The score of critical appraisal, determined by the critical appraisal skills program cohort study checklist, is shown in percentages

Author	Aim	Statistical method	Results	Conclusion	CAPS
Leiderman et al. 1994^14^	Examine the time course of alterations in glucose metabolism in relation to the interval from the last seizure and seizure type	Absolute measurement (statistical comparison ND)	FIAS: LCMRglc in the inferior temporal region was significantly higher in 24‐ to 48‐h period than >48 h period (0.746 vs 0.674, *P* = .02). FAS: LCMRglc was highest in <24 h period, dropped 40% from <24 h period to the 24‐ to 48‐h period (0.883 vs 0.523, *p* = .02). The LCMRglc raised 45% from 24‐48 period to >48 period (0.0.523 vs 0.760, *p* = .06).	The time course of metabolism was different for the two types of seizures. The results suggest that the hypermetabolic effects of a FIAS appear to persist for at least 48 h and for SPS for 24 h. 24‐48 h after FAS showed the strongest hypometabolic rate compared to contralateral region.	92%
Savic et al. 1997^17^	Relate the degree and extent of extra focal hypometabolism to the time between the performance of the PET scan and the preceding seizure	Pearson’s correlation	FIAS: No significant correlation between time from last seizure to extra focal hypometabolism (*r* = .1; *P* = .6) or the number of hypometabolic ROIs (*r* = .1; *P* = .7)	Time between seizure and performance of PET scan did not correlate with the degree or extent of hypometabolism.	50%
Gaillard et al. 2007^18^	Study the evolution of cerebral glucose metabolism after focal onset seizure in children, and its relation to clinical variables	ND	The mean seizure number was higher in the year before an abnormal scan (44.9 ± 64.3 vs 15.2 ± 15.1; *P* < .02). The time since the last seizure was shorter before an abnormal scan (93.8 ± 183.6 days vs 290.4 ± 347 0.4 days; *P* < .01)	Serial FDG‐PET is affected by seizure frequency and time since last seizure	55%
Tepmongkol et al. 2013^20^	Factors affecting BTH in unilateral medial TLE	Fisher’s exact test	Duration ≤2 d from the last seizure to the PET scan showed a significant effect on the finding of BTH (*P* = .036).	PET needs to be performed at least 2 d after the last seizure and should be rescheduled if the duration from the last seizure to the PET scan is less than 2 d.	91%
		Multivariate analysis	Time was an independent factor to predict BTH (OR 15.17 [CI: 1.50–153.18]; *P* = .021)		
Kumar et al. 2010^19^	To test and optimize the performance of SPM analysis	Correlated or ANOVA test	Time between PET and last seizure showed no correlation with sensitivity or specificity of either visual analysis or SPM analyses.	No correlation between clinical variables and FDG‐PET analysis.	20%

Abbreviations: BTH, bilateral temporal lobe hypometabolism; CI, confidence interval; FAS, Focal Aware Seizure (formerly: simple partial seizure); FDG‐PET, fluoro‐2‐deoxyglucose positron emission tomography; FIAS, Focal Impaired Awareness Seizure (formerly: complex partial seizure); LCMRglc, local cerebral metabolic rate for glucose; ND, not documented; OR, odds ratio; PET, positron emission tomography; ROI, regions of interest; SPM, Statistical Parametric Mapping.

The datasets in the reviewed papers are described rather sparsely. Apart from the data given in Table S3, no additional insights into the datasets have been reported. The most frequent determinant was “time since last seizure”; hence, this term was used in this systematic review. Other terms included: “duration in days from the last seizure to PET” and “interval since the last seizure.”

### 3.3 Critical appraisal

The quality scoring overview is enclosed in Table S5. Additional explanation is added.

Leiderman et al’s study^14^ (CAPS 92%) was the only study with dynamic FDG‐PET imaging, resulting in quantitative data for the time course of metabolic change. They distinguished between simple and complex partial seizures. A drawback of this study is that they did not report standard deviations with the mean metabolic glucose measurements. Therefore, outliers and individual differences are unknown.

Savic et al^17^ (CAPS 50%) concluded that there was no correlation between time since last seizure and the extent of hypometabolism. They searched for a linear correlation under the assumption of normal data distribution, which is doubtful because only an average of 2.5 days was presented without a range or standard deviation. Furthermore, for a linear correlation, multiple data points on a timeline are needed. The period in this study was too short to provide this.

Gaillard et al^18^ (CAPS 55%) did not document time point measurements and which statistical analysis they used. Their FDG‐PET protocol was unclear. Their database included multiple FDG‐PET scans from the same patient with potentially interesting findings; unfortunately, results were not documented per patient. No multiple regression analysis was conducted to assess the independent contribution of seizure frequency and time since last seizure.

Tepmongkol et al^20^ (CAPS 91%) described a population with generalized seizures only; their conclusion may therefore not apply to other seizure types. They conducted univariate and multivariate analyses determining individual or combined contribution of determinants to the outcome.

Kumar et al^19^ (CAPS 20%) did not focus on the determinant of this systematic review, which is time since last seizure. Measurement of time points was not documented. In the results, no correlation was said to be found, but it is unclear which statistical analysis they used and no *P*‐values were reported. Paired *t*‐test, unpaired *t*‐test, and ANOVA were used, which implies an assumption of normally distributed data. With a range from 1 to 90 days and a median of 1.5 days, this is impossible. They did not report seizure type which may be a confounder.

## 4 DISCUSSION

We wanted to know when FDG‐PET may be safely interpreted on the assumption of interictal hypometabolism of the epileptic focus, using clinical time since last seizure. We identified and critically appraised five studies with a total of 138 subjects. We focused on temporal differences in FDG‐PET rather than spatial differences.

Three out of five studies reported that time since last seizure significantly influences results. The studies which reported no correlation were of low quality. The two high‐quality studies solely included adult patients with TLE. A safe and practical advice may be formulated: acquisition of FDG‐PET for TLE should ideally be performed at least 1 day after a focal aware seizure and 2 days with impaired awareness or a focal to bilateral tonic–clonic seizure.

Often patients do not know when they had a seizure. Probably only about 50% of seizures are reported by patients and caregivers.^21^ EEG during FDG‐PET acquisition is recommended, but it is difficult to define a postictal state in EEG and lacks to provide information about the hours preceding the FDG‐PET. Subclinical seizures may, however, show up during FDG‐PET acquisition, especially in children, that may also confound FDG‐PET.^22^, ^23^ Several other studies concluded that frequent or continuous seizures are associated with hypermetabolism on FDG‐PET.^12^, ^22^, ^23^ A high seizure frequency will raise the odds of a shorter time interval between seizure and FDG‐PET imaging. We believe that the interpretation of FDG‐PET in patients suffering from frequent seizures as well as a recent seizure should be extra careful. Ideally, continuous EEG monitoring starting 2 hours before the injection up to 20 min after the injection should be performed for detection of epileptic activity to avoid false interpretation; however, this is not always practically feasible.

The recommendation may not apply to children. Gaillard et al. did conclude that serial FDG‐PET in children is affected by time since last seizure and seizure frequency.^18^ Unfortunately, data supplied in their article preclude a recommendation. In another study, children were excluded when the last seizure occurred less than 24 hours before FDG‐PET imaging.^24^

The duration of the metabolically postictal phase has been rarely researched and should be a topic for future studies. Studies of postictal change in perfusion do not necessarily translate to metabolism.^25^, ^26^ Other interesting questions concerning PET imaging in epilepsy are among others quantitative post‐processing increasing the diagnostic value of the PET^27^ and novel PET ligands such as ones targeting mediators of the immune system. These topics definitely require investigation but are beyond the scope of this clinically focused review.

Dynamic ictal FDG‐PET would give the opportunity to picture the metabolic transition from the postictal to the interictal phase and add context to individual differences.^28^

## 5 CONCLUSION

Only five studies have researched the effect of time since last seizure on brain metabolism measured with FDG‐PET in patients with epilepsy. These suggest that in adults, brain glucose metabolism in focal seizures will take at least 24 hours to return to its hypometabolic baseline. In focal to bilateral seizures, a minimum of 48 hours is needed. FDG‐PET imaging performed within 24‐48 hours can result in relative hypermetabolism of the epileptic focus compared to the contralateral (healthy) hemisphere and result in false lateralization. Further research on this topic is advised.

## CONFLICT OF INTEREST

None of the authors has any conflict of interest to disclose. We confirm that we have read the Journal’s position on issues involved in ethical publication and affirm that this report is consistent with those guidelines.

## Supporting information


Supplementary Matrials S1‐S5

## References

[1] Cendes F , Theodore WH , Brinkmann BH , Sulc V , Cascino GD . Neuroimaging of epilepsy. Handb Clin Neurol. 2016;136:985–1014. 10.1016/B978-0-444-53486-6.00051-X 27430454 PMC5256664

[2] Theodore WH . When is positron emission tomography really necessary in epilepsy diagnosis? Curr Opin Neurol. 2002;15(2):191–5. 10.1097/00019052-200204 000-00011 11923634

[3] Uijl SG , Leijten FSS , Arends JBAM , Parra J , Van Huffelen AC , Moons KGM . The added value of [18F]‐fluoro‐D‐deoxyglucose positron emission tomography in screening for temporal lobe epilepsy surgery. Epilepsia. 2007;48(11):2121–9. 10.1111/j.1528-1167.2007.01197.x 17651417

[4] Theodore WH . Cerebral blood flow and glucose metabolism in human epilepsy. Adv Neurol. 1999;79:873–81. 10514870

[5] Meltzer CC , Adelson PD , Brenner RP , Crumrine PK , Cott A , Schiff DP , et al. Planned ictal FDG PET imaging for localization of extratemporal epileptic foci. Epilepsia. 2000;41(2):193–200. 10.1111/j.1528-1157.2000.tb00139.x 10691116

[6] Chugani CD , Chugani HT . Basic mechanisms of childhood epilepsies: studies with positron emission tomography. Adv Neurol. 1999;79(883):91. 10514871

[7] Koutroumanidis M , Binnie CD , Elwes RDC , Polkey CE , Seed P , Alarcon G , et al. Interictal regional slow activity in temporal lobe epilepsy correlates with lateral temporal hypometabolism as imaged with 18FDG PET: neurophysiological and metabolic implications. J Neurol Neurosurg Psychiatry. 1998;65(2):170–6. 10.1136/jnnp.65.2.170 9703166 PMC2170184

[8] Fisher M , Scharman RS , deCurtis HE . How can we identify ictal and interictal abnormal activity? Adv Exp Med Biol. 2014;813:3–23. 10.1007/978-94-017-8914-1 25012363 PMC4375749

[9] Pottkämper JCM , Hofmeijer J , van Waarde JA , van Putten MJAM . The postictal state — what do we know? Epilepsia. 2020;61(6):1045–61. 10.1111/epi.16519 32396219 PMC7317965

[10] Nooraine J , Iyer RB , Raghavendra S . Ictal PET in presurgical workup of refractory extratemporal epilepsy. Ann Indian Acad Neurol. 2013;16(4):676–7. 10.4103/0972-2327.120475 24339606 PMC3841627

[11] Olson DM , Chugani HT , Shewmon DA , Phelps ME , Peacock WJ . Electrocorticographic confirmation of focal positron emission tomographic abnormalities in children with intractable epilepsy. Epilepsia. 1990;31(6):731–9. 10.1111/j.1528-1157.1990.tb05514.x 2245803

[12] Struck AF , Westover MB , Hall LT , Deck GM , Cole AJ , Rosenthal ES . Metabolic correlates of the ictal‐interictal continuum: FDG‐PET during continuous EEG. Neurocrit Care. 2016;24(3):324–31. 10.1007/s12028-016-0245-y 27169855 PMC5478419

[13] Decoo D , Destée A . PET studies in epilepsy. Acta Neurol Belg. 2015;97(3):196–9. 9345593

[14] Leiderman DB , Albert P , Balish M , Bromfield E , Theodore WH . The dynamics of metabolic change following seizures as measured by positron emission tomography with Fludeoxyglucose F18. Arch Neurol. 1994;51:932–6. 8080394 10.1001/archneur.1994.00540210106019

[15] Critical Appraisal Skills Programme . CASP Cohort Study Checklist. 2018. https://casp‐uk.net/casp‐tools‐checklists/. Accessed on January 8, 2020. CASP Checklist: Cohort Study. www.casp‐uk.net

[16] Mann CJ . Observational research methods. Research design II: cohort, cross sectional, and case‐control studies. Emerg Med J. 2003;20:54–61. 10.1016/j.afjem.2011.12.004 12533370 PMC1726024

[17] Savic I , Altshuler L , Baxter L , Engel J . Pattern of interictal hypometabolism in PET scans with fludeoxyglucose F 18 reflects prior seizure types in patients with mesial temporal lobe seizures. Arch Neurol. 1997;54(2):129–36. 10.1001/archneur.1997.00550140011006 9041853

[18] Gaillard WD , Weinstein S , Conry J , Pearl PL , Fazilat S , Fazilat S , et al. Prognosis of children with partial epilepsy: MRI and serial 18FDG‐PET. Neurology. 2007;68(9):655–9. 10.1212/01.wnl.0000255942.25101.8d 17325271

[19] Kumar A , Juhász C , Asano E , Sood S , Muzik O , Chugani HT . Objective detection of epileptic foci by18F‐FDG PET in children undergoing epilepsy surgery. J Nucl Med. 2010;51(12):1901–7. 10.2967/jnumed.110.075390 21078805 PMC3157889

[20] Tepmongkol S , Srikijvilaikul T , Vasavid P . Factors affecting bilateral temporal lobe hypometabolism on 18F‐FDG PET brain scan in unilateral medial temporal lobe epilepsy. Epilepsy Behav. 2013;29(2):386–9. 10.1016/j.yebeh.2013.08.017 24074882

[21] Elger CE , Hoppe C . Diagnostic challenges in epilepsy: seizure under‐reporting and seizure detection. Lancet Neurol. 2018;17(3):279–88. 10.1016/S1474-4422(18)30038-3 29452687

[22] Schur S , Allen V , White A , Mirsky D , Stence N , O'Neill B , et al. Significance of FDG‐PET hypermetabolism in children with intractable focal epilepsy. Pediatr Neurosurg. 2018;53(3):153–62. 10.1159/000487088 29672310

[23] Chugani HT , Shewmon DA , Khanna S , Phelps ME . Interictal and postictal focal hypermetabolism on positron emission tomography. Pediatr Neurol. 1993;9(1):10–5. 10.1016/0887-8994(93)90003-U 8452593

[24] Zhu Y , Feng J , Wu S , Hou H , Ji J , Zhang K , et al. Glucose metabolic profile by visual assessment combined with statistical parametric mapping analysis in peDiatric patients with epilepsy. J Nucl Med. 2017;58(8):1293–9. 10.2967/jnumed.116.187492 28104740

[25] Tatlidil R . Persistent postictal hyperperfusion demonstrated with PET. Epilepsy Res. 2000;42(2–3):83–8. 10.1016/S0920-1211(00)00135-2 11074180

[26] Kovács R , Gerevich Z , Friedman A , Otáhal J , Prager O , Jimenez‐Mateos EM . Bioenergetic mechanisms of seizure control. Front Cell Neurosci. 2018;12:1–14. 10.3389/fncel.2018.00335 30349461 PMC6187982

[27] Lammertsma AA . Forward to the past: the case for quantitative PET imaging. J Nucl Med. 2017;58(7):1019–24. 10.2967/jnumed.116.188029 28522743

[28] Tang Y , Liow J , Zhang Z , Li J , Long T , Li Y . The evaluation of dynamic FDG‐PET for detecting epileptic foci and analyzing reduced glucose phosphorylation in refractory epilepsy. Front Neurosci. 2019;12:1–11. 10.3389/fnins.2018.00993 PMC6333859 30686968

