# Supplementary material for: Optimal timing of interictal FDG‐PET for epilepsy surgery: A systematic review on time since last seizure
Source: Epilepsia Open. 2022 Jun 20;7(3):512–7. doi: 10.1002/epi4.12617 (PMC9436292; doi:10.1002/epi4.12617)
Supplement: Supplementary file 1 — Supplementary Matrials S1‐S5 [file EPI4-7-512-s001.docx]

Appendix S1

Search string
The following Mesh Terms were used in PubMed: Epilepsy, seizures, fluorodeoxyglucose F18 and brain/Metabolism. No filter was applied, the search was not constrained on publication year. Used Emtree terms in Embase were: Epilepsy, seizure, Fluorodeoxyglucose F18, and Brain metabolism. For all terms, different spelling and synonyms were used. In Embase the sources filter was applied to only include Embase results and exclude Medline because they overlap with results in PubMed. Other filters were not applied in the search.

Full electronic search strategy for PubMed:
*("epilepsy"[MeSH Terms] OR "epilepsy"[Title/Abstract] OR "epileptic foci" [Title/Abstract] OR "epilep*"[Title/Abstract]) AND ("seizures"[Mesh Terms] OR "seizur*"[Title/Abstract]) AND ("time"[Title/Abstract] OR "duration"[Title/Abstract]) AND ("Fluorodeoxyglucose F18"[Mesh Terms] OR "Fluorodeoxyglucose F18"[Title/Abstract] OR "fluorodeoxyglucose"[Title/Abstract] OR "FDG-PET"[Title/Abstract] OR "fluorodeoxyglucose-PET"[Title/Abstract] OR "18F FDG-PET"[Title/Abstract] OR "fludeoxyglucose"[Title/Abstract] OR "fluorine 18"[Title/Abstract] OR "F18"[Title/Abstract] OR "18F"[Title/Abstract]) AND ("Brain/metabolism"[Mesh] OR "hypometabolism"[Title/Abstract] OR "metaboli*"[Title/Abstract] OR "hypometabol*"[Title/Abstract])*

Search strategy for Embase:

*(('epilepsy'/exp) OR ('epilepsy':ti,ab,kw) OR ('epileptic foci':ti,ab,kw) OR ('epilep*':ti,ab,kw)) AND (('seizure'/exp) OR ('seizur*':ti,ab,kw)) AND (('time':ti,ab,kw) OR ('duration':ti,ab,kw)) AND (('Fluorodeoxyglucose f 18'/exp) OR ('Fluorodeoxyglucose F18':ti,ab,kw) OR ('fluorodeoxyglucose':ti,ab,kw) OR ('FDG-PET':ti,ab,kw) OR ('fluorodeoxyglucose-PET':ti,ab,kw) OR ('18F FDG-PET':ti,ab,kw) OR ('fludeoxyglucose':ti,ab,kw) OR ('fluorine 18':ti,ab,kw) OR ('F18':ti,ab,kw) OR ('18F':ti,ab,kw)) AND (('brain metabolism'/exp) OR ('hypometabolism':ti,ab,kw) OR ('metaboli*':ti,ab,kw) OR ('hypometabol*':ti,ab,kw))*

Figure S2


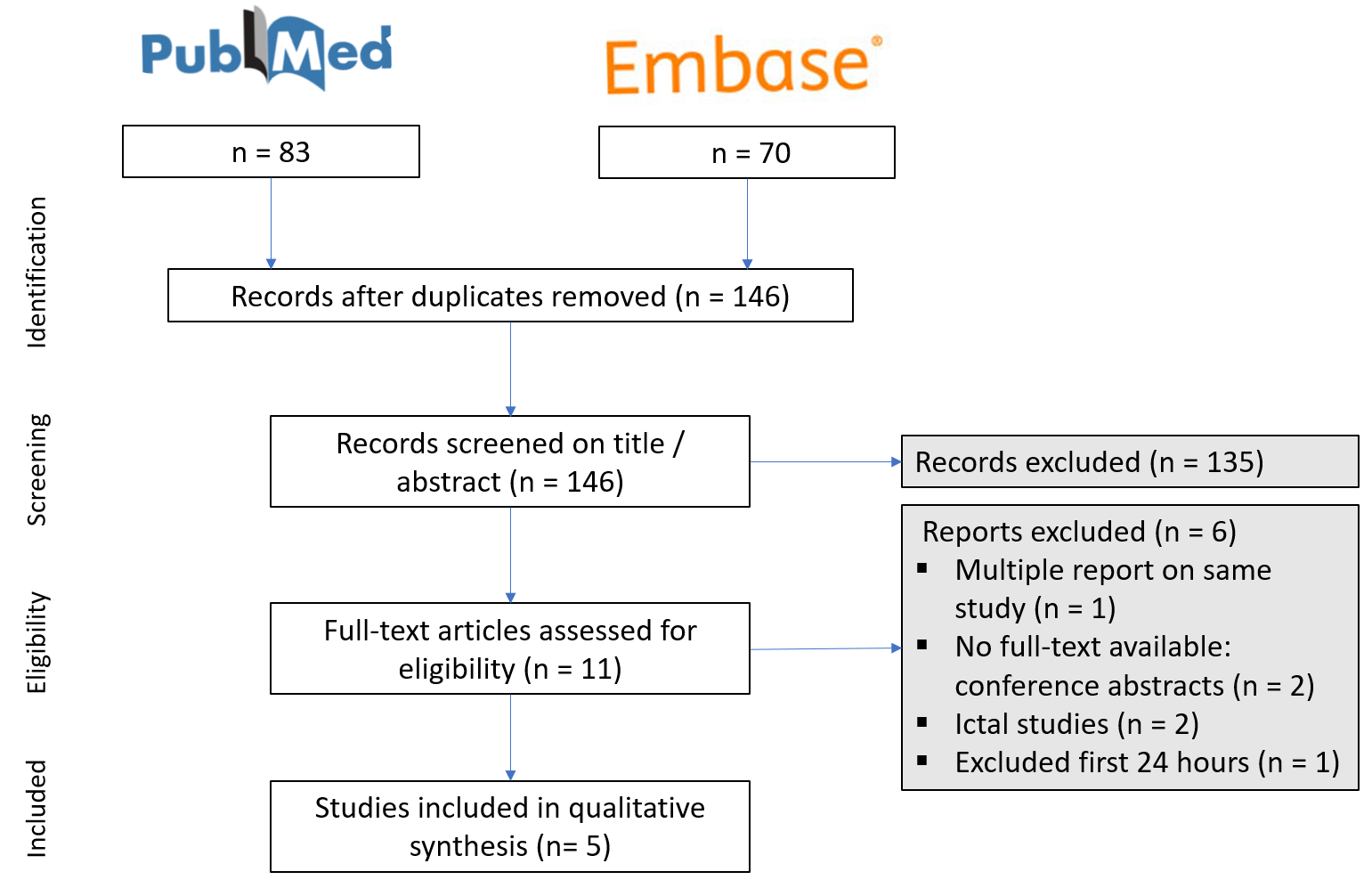


Figure 2 Flow diagram of the systematic review process

Table S3

Table S2 Study characteristics. The colored rectangle surrounding author corresponds with the critical appraisal score. From left to right; Design: R = retrospective or P = prospective cohort. Population: N = number, C = Children, A = Adult. Time points: (d,m,y) = day, month, year. Data of time points: Co = continuous or Ca = categorical. FDG-PET analysis: a,b,c,d,e corresponds with outcome measurements of FDG-PET analysis, see supporting information S3.

| Author |  | Design | | Population | | | | | Method | | | |
| --- | --- | --- | --- | --- | --- | --- | --- | --- | --- | --- | --- | --- |
|  | Year | R | P | N | C | A | Inclusion | Seizure type | Time points (d,m,y) | Co | Ca | FDG-PET |
| Leiderman et al. [10] | 1994 |  | x | 32 |  | x | Medically intractable inferior temporal epilepsy and lateralized seizure onset | Complex partial seizure (CPS) n=25, simple partial (SPS) n=16, generalized tonic-clonic (GTCS) n=10 | 3 categories:  <24h, 24-48h and >48h |  | x | d |
| Savic et al. [14] | 1997 | x |  | 53 |  | x | TLE and location congruent to seizures | Complex partial seizures (CPSs) | Days (mean 2,5 days no SD given) | x |  | b.i.ii |
| Gaillard et al. [15] | 2007 | x |  | 38 | x |  | At least 3 partial seizures before their first FDG-PET (multiple FDG-PET scans) | Unprovoked partial seizure | ND | x |  | b,e |
| Kumar et al. [16] | 2010 | x |  | 20 | x |  | Intractable focal epilepsy who were seizure free after surgery | ND | Days, median 1,5d (range 1-90d) | x |  | a, c |
| Tepmongkol et al. [17] | 2013 | x |  | 40 |  | x | Intractable medial TLE with unilateral temporal ictal onset | Generalized tonic seizure and generalized tonic-clonic seizure | 2 categories:  ≤ 2d or ≥ 2d |  | x | a.i |
| Abbreviations: TLE = Temporal Lobe Epilepsy, ND = Not documented in article, SD = standard deviation, CPS = complex partial seizure (now: focal impaired awareness seizure), SPS = simple partial seizure (now: focal aware seizure), GTCS = generalized tonic-clonic (now: focal to bilateral tonic clonic seizure), FDG-PET = fluoro-2-deoxyglucose positron emission tomography. | | | | | | | | | | | | |

Appendix S4

**Outcome measurements of FDG-PET analysis**
The clinical outcomes from FDG-PET were obtained differently in the included studies. Firstly, different methods for data extraction from FDG-PET were used. These methods are summarized below (a – e). Secondly, different definitions of abnormal glucose metabolism were used (i – ii).

1. *Visual inspection:* The images were visually evaluated by two nuclear medicine specialists blinded for clinical data. They evaluated and reported separately and discordant results were reviewed until consensus was reached. Interpretation usually involves assessing left-right asymmetries in activity concentration (AC) or standardized uptake value (SUV). This is a relative measurement derived from a static PET scan.
   1. *Hypometabolism was defined as an area with a 20% or lower metabolism compared to other areas of the cortices.* *The hypometabolism of temporal lobe(s) was categorized into 2 patterns: unilateral temporal hypometabolism (UTH) and bilateral temporal hypometabolism (BTH).*
2. *Region of Interests (ROIs):* ROIs were drawn using a standardized template as a guide to localization. ROIs were manually adjusted by a clinician blinded to clinical data.
   1. A total number of abnormal ROIs per patient was calculated. Abnormal was defined as ratios outside 2 standard deviations (SD) of control subjects. The dimensionless ratio was determined for epileptogenic ROI divided by the average cortical value from the contralateral (non-epileptogenic) hemisphere, see **equation (2).**
      $Ratio = \left[ \frac{ROIx}{ROIcortex} \right]_{patient} (2)$
   2. Mean percentage reductions were calculated within and outside the epileptogenic zone according to **equation** **(3).**
$Mean \% =\left( \frac{\left[ \frac{ROIx}{ROIcortex} \right]_{control} - \left[ \frac{ROIx}{ROIcortex} \right]_{patient}}{\left[ \frac{ROIx}{ROIcortex} \right]_{control}} \right)* 100 (3)$
3. *Statistical parametric mapping (SPM):* is a free and open source software for objective voxel-based analysis technique to detect focal abnormalities corresponding to seizure foci [19]. The software starts with a spatial transformation to realign PET images in a standard anatomical space, finding the most significant differences between functional PET and anatomical MRI. An age-matched healthy control group is needed.
4. *Quantitative analysis:* quantitative measurement of absolute glucose metabolism. Blood sampling is required in combination with dynamic PET scanning. Value expressed as local cerebral metabolic rate for glucose (LCMRglc).
5. *Semiquantitative analysis:* ROIs measurements or SPM are used to calculate an absolute asymmetry index (|AI|), see **equation (4).**

$$|AI|=\frac{left - right}{(left+right)/2} (4)$$

Table S5

*Table S4. Scoring overview of included articles according to questions from the Critical Appraisal Skills Programme (CASP) tool for cohort studies.*

|  | **Q1** | **Q2** | **Q3** | **Q4** | **Q5** | | **Q6** | | **Q7** | | **Q8** | | **Q9** | | **Q10** | | **Q11** | | **Q12** | |  | |
| --- | --- | --- | --- | --- | --- | --- | --- | --- | --- | --- | --- | --- | --- | --- | --- | --- | --- | --- | --- | --- | --- | --- |
| **Author** | **Clearly focused issue** | **Recruitment cohort** | **Measurement determinant** | **Measurement outcome** | **Confounding factors** | | **Quality follow-up** | | **Reporting results** | | **Precision results (p-value)** | | **Plausibility results** | | **Applicability results** | | **Fit of results w/ evidence** | | **Practical implications** | | **Total score** | |
| **Tepmongkol et al.** [17] | 1 | 1 | 1 | 0 | 1 | | N/A | | 1 | | 1 | | 1 | | 1 | | 1 | | 1 | | 91% | |
| Savic et al. [14] | 1 | 1 | 1 | 1 | 0 | | N/A | | 0 | | 1 | | 0 | | N/A | | 0 | | 0 | | 50% | |
| Kumar et al. [16] | 0 | 1 | 1 | 1 | 0 | | N/A | | 0 | | 0 | | 0 | | N/A | | 0 | | 0 | | 20% | |
| Gaillard et al. [15] | 1 | 1 | 0 | 1 | 0 | | N/A | | 0 | | 1 | | 1 | | 1 | | 0 | | 0 | | 55% | |
| Leiderman et al. [10] | 1 | 1 | 1 | 1 | 1 | | 1 | | 0 | | 1 | | 1 | | 1 | | 1 | | 1 | | 92% | |
|  |  |  |  |  |  | |  | |  | |  | |  | |  | |  | |  | |  | |
| Scoring System: |  |  |  |  |  | |  | |  | |  | |  | |  | |  | |  | |  | |
| 1 = if the answer to the question is *yes* | | | | | |  | |  | |  | |  | |  | |  | |  | |  | |  | |
| 0 = if the answer to the question is *no* | | | | | |  | |  | |  | |  | |  | |  | |  | |  | |  | |
| N/A = Not Applicable | | | | | |  | |  | |  | |  | |  | |  | |  | |  | |  | |
| $Total score= \frac{Total questions answered with yes}{Total answered questions} \times100\%$ (1) | | | | | | | | | | | | | |  | |  | |  | |  | |  |
| Additional information on specific questions: **Q2:** Because all studies except one are retrospective studies, the selection bias due to specific referral patterns cannot be avoided entirely. All studies still received one point in the critical appraisal because the possible selection bias did not comprise the generalizability of the findings.  **Q4:** Visual inspection was defined as a subjective measurement of outcome and scored as zero. **Q5:** Confounding factors were only scored as 1 when type of seizure was included and multivariate linear regression analysis was performed to determine independent contribution of individual variables. **Q6:** Quality of follow up was not applicable (N/A) when the cohort selection included a specific follow up. | | | | | | | | | | | | | | | | | | | | | | |
